# Acceptability of the live biotherapeutic LACTIN-V (*Lactobacillus crispatus* CTV-05) among young women at high risk of HIV acquisition in South Africa: data from the phase 2 placebo-controlled trial

**DOI:** 10.3389/frph.2025.1544458

**Published:** 2025-03-25

**Authors:** A. Hemmerling, V. Govender, K. Dong, M. Dong, Sooseela Pillay, T. Ndung’u, A. Bhoola, J. Moodley, G. Casillas, L. Lagenaur, C. M. Mitchell, D. S. Kwon, C. R. Cohen

**Affiliations:** ^1^ Department of Obstetrics, Gynecology and Reproductive Sciences, University of California, San Francisco, CA, United States; ^2^ The Aurum Institute, Johannesburg, South Africa; ^3^ Ragon Institute of Mass General, MIT and Harvard, Cambridge, MA, United States; ^4^ Infectious Diseases Division, Massachusetts General Hospital, Boston, MA, United States; ^5^ Department of Medicine, Harvard Medical School, Boston, MA, United States; ^6^ Females Rising Through Education, Support and Health, Umlazi, South Africa; ^7^ HIV Pathogenesis Programme, the Doris Duke Medical Research Institute, University of KwaZulu-Natal, Durban, South Africa; ^8^ Africa Health Research Institute, Durban, South Africa; ^9^ Division of Infection and Immunity, University College London, London, United Kingdom; ^10^ Osel, Inc., Mountain View, CA, United States; ^11^ Department of Obstetrics & Gynecology, Massachusetts General Hospital, Boston, MA, United States; ^12^ Department of Obstetrics, Gynecology & Reproductive Biology, Harvard Medical School, Boston, MA, United States

**Keywords:** lower genital tract infections, prevention of HIV, vaginal microbiota, bacterial vaginosis, live biotherapeutic products, probiotics

## Abstract

**Introduction:**

Live biotherapeutic products (LBPs) containing *Lactobacillus crispatus* may optimize the vaginal microbiota, reduce genital inflammation, and protect against HIV acquisition. Determining acceptability of LBPs among African women at high risk of HIV is essential to guide product development.

**Methods:**

The phase 2 double-blind randomized placebo-controlled trial recruited young sexually active cis-women with vaginal dysbiosis from a community-based research clinic. Following antibiotics (oral metronidazole), participants were randomized (2:1) to receive 11 doses of LACTIN-V (2 × 10^9^ L*. crispatus* CTV-05) or placebo over 4 weeks. A questionnaire assessed product acceptability.

**Results:**

Forty-five young Black South African women were randomized to LACTIN-V (*N* = 32) or placebo (*N* = 13). Forty-two (93.3%) had an active sexual partner. Adherence was high with 36 participants (80.0%) completing all 11 doses. Of the 43 participants who completed the acceptability questionnaire, 38 (88.4%) were satisfied using the vaginal applicator and 41 (95.5%) confirmed ease of use. For 14 (32.5%) participants, product use without the partner knowing was important. Thirty-one (72.1%) participants felt that partner approval for product use was not important. On Likert scales of 0–10 (lowest to highest), agreement with positive product attributes (effective, comfortable, easy to use) scored at means of  ≥6.7. Negative product attributes (dosing, leakage, vaginal dryness, partner's disapproval) were rated less important with lower mean scores ≤3.2. Overall, 75% of participants would use the product again, with no significant difference between study arms.

**Conclusions:**

Young South African women at high risk of HIV found the LACTIN-V study product highly acceptable and easy to use.

**Clinical Trial Registration:**

[clinicaltrials.gov], identifier [NCT05022212].

## 1 Introduction

Novel approaches to reduce HIV acquisition in women are needed, and attention has turned to optimizing the vaginal microbial community and strengthening its defense mechanisms. Previous research has found that women with a high prevalence of *Lactobacillus crispatus* have a lower risk for inflammation and HIV acquisition (1, 2). A healthy vaginal microbial community is dominated by lactobacilli. Bacterial vaginosis (BV), a form of vaginal dysbiosis caused by an overgrowth of pathogens, affects 15%–50% of women of reproductive age (3). BV is associated with genital inflammation in women (4–6), and is implicated in increased risk of HIV acquisition (1, 2). Additionally, BV increases risk for preterm birth (PTB) (7), human papilloma virus (HPV) persistence and cervical dysplasia (8). For decades, available BV treatments have been limited to repeat courses of antibiotics, with modest improvement and high recurrence rates (9, 10).

Live biotherapeutic products (LBPs)—a term introduced by the Food and Drug Administration (FDA) in 2012—are biological products evaluated as drugs for safety and efficacy to prevent, treat or cure disease. Vaginal LBPs are designed to establish colonization with human vaginal *Lactobacillus* strains, reduce genital inflammation and protect against adverse reproductive outcomes. LACTIN-V, a vaginally delivered LBP developed by Osel Inc. (Mountain View, CA, USA) containing the endogenous human vaginal strain of *Lactobacillus crispatus* CTV-05, is currently being evaluated for preventing recurrent (r)BV, PTB (11), female HIV acquisition and the prevention of cervical cancer. To date, over 900 women have participated in clinical studies assessing LACTIN-V. Results have demonstrated an excellent safety profile, sustainable vaginal colonization of the CTV-05 strain, and effectiveness for the prevention of rBV (12–14). Acceptability of LACTIN-V in the phase 2b clinical trial conducted in the U.S. was high; 86% of participants were satisfied with the product, and 84% would use it if available in the future (14).

While data on the acceptability of a vaginal LBP among African women are scarce (11, 15, 16), evidence evaluating other vaginally administered drug products for HIV prevention have demonstrated that products of poor acceptability and low adherence can result in a lack of effectiveness (17–19). These findings underscore the importance of assessing product acceptability early in drug development among all target populations. Therefore, as part of a double-blind randomized placebo-controlled phase 2 clinical trial of LACTIN-V in South Africa we sought to measure the acceptability of this first vaginally administered LBP using a *L. crispatus* strain among young South African women at high risk of HIV acquisition.

## 2 Materials and methods

Between May 2021 and April 2023, cis-women were recruited from the FRESH (Females Rising through Education, Support and Health) study in Umlazi, KwaZulu-Natal, South Africa for this study to assess the impact of LACTIN-V to increase vaginal abundance of *L. crispatus* and decrease genital tract inflammation associated with increased HIV acquisition. Ethical approval was obtained at all participating institutions, and written informed consent was obtained prior to participant enrollment. We obtained approval from the South African Health Products Regulatory Authority (# 20190519) and registered the study on clinicaltrials.gov (NCT05022212).

Women were eligible to participate if they were 18–23 years of age, not pregnant, enrolled in the parent FRESH study, diagnosed with a Nugent score of 4–10 on vaginal Gram Stain, with negative STI test results including HIV, on long-acting contraception, and willing to abstain from using other vaginal products. They were ineligible if deep epithelial disruption was observed on genital examination at screening, they had recently taken antibiotic or antifungal medication or other investigational drugs as part of a research study, had a recent IUD insertion/removal or pelvic surgery, or were using a vaginal ring. Participants with vaginal dysbiosis (diagnosed as Nugent Score 4–10 on Gram stain) at baseline received oral 400 mg metronidazole twice daily over 7 days, the standard of care for BV treatment in South Africa. Within 48 h of completing at least 12 doses of antibiotic treatment, 45 participants were randomized (2:1) to receive 11 doses of LACTIN-V (2 × 10^9^ L*. crispatus* CTV-05) or matched placebo over 4 weeks (5 doses during week 1, then twice weekly). LACTIN-V is a lyophilized (stabilized) dry powder containing the CTV-05 *Lactobacillus* strain and a proprietary inert nutrient matrix. The matched placebo only contained the nutrient matrix and was indistinguishable from the applicators containing LACTIN-V. Participants were trained by the study nurse to self-administer the study product (LACTIN-V or placebo). A prefilled vaginal applicator is inserted to deliver the powder into the upper vaginal vault where it adheres to the epithelium and rehydrates. Participants were asked to abstain from sexual intercourse for 12 h after study product administration to ensure the product would remain inside the vagina. Participants returned to the clinic twice weekly as part of their normal routine visits within the FRESH study. At each visit, they administered their study product from their assigned kits stored on site, and reported on adverse events, menstruation and sexual behavior. Eight of the 11 doses were administered in the clinic during the twice weekly visits, while the additional three doses during Week 1 were administered at home. Follow up visits at 4 and 8 weeks included a gynecological exam and sample collection of vaginal swabs, cervical swabs, cervicovaginal lavages (CVL) and endocervical cytobrushes to assess microbial composition, inflammatory cytokines/chemokines, and endocervical HIV target cells. A more detailed description of study procedures has been published elsewhere in a companion manuscript (20).

The original sample size was based on the main study objective of detecting decreased inflammation. With a target sample size of 60 women in a 2:1 randomization there would have been 88% power to detect a 40% absolute difference in the proportion of women with decreased inflammation between the two arms. Several delays of the study due to COVID and later damage to the clinic site during local social unrest, the intended sample size was reduced from 60 to 45 participants, still achieving adequate power (>80%) to detect effect sizes of 50%.

The study included a questionnaire evaluating product acceptability that was administered by study staff at the end of study product use (4 weeks). The non-validated questionnaire had been slightly adapted from a version previously used during the LACTIN-V phase 2a and 2b studies, and contained multiple choice questions on their experience with the study product, as well as exploring support by sexual partners. It also assessed positive product attributes (whether participants felt that the study product was effective, comfortable, and easy to use) and negative product attributes (frequency or timing of use, vaginal dryness, messiness or leakage, or partner's disapproval) on Likert Scales 0–10. Additionally, collected data on participants’ demographics, gynecological history and sexual behavior, use of other vaginal products were included. Adherence was defined as administrating at least 9 of 11 doses of study product, of which eight were administered during clinic visits and three self-reported as administered at home, limiting dependance on self-reported adherence. Participants and study staff remained blinded as to their placement in the LACTIN-V or placebo group when they completed the acceptability questionnaire. Safety analyses were conducted with the Intent to Treat (ITT) population. Confidence intervals for AE rates were estimated using methods for exact binomial confidence intervals. For demographic data, Wilcoxon rank-sum and Fisher's exact tests were respectively used to compare continuous and categorical participant characteristics between the two arms. *P*-values are two sided and confidence intervals are at the 95% level. For acceptability results, we present descriptive statistics overall using simple proportions and stratified by intervention and placebo groups using SAS® software, version 9.04.

## 3 Results

Among 45 Black South African women randomized to LACTIN-V (*N* = 32) or placebo (*N* = 13), the median age was 21. Thirty-one (68.9%) completed Grade 12 (high school/matric), of whom seven (15.6%) participants went on to enroll in tertiary education with a median of three years, while 12 (26.7%) had completed Grade 11 and two participants (4.4%) completed Grade 10 (Table 1).

**Table 1:** Summary of demographics by treatment group.

*n* (%)	LACTIN-V (*N* = 32)	Placebo (*N* = 13)	All
Sex			
Female	32 (100)	13 (100)	45 (100)
Race			
Black or African	32 (100)	13 (100)	45 (100)
Age (years)			
*n*	32	13	45
Mean	21.3	20.4	21.1
Standard deviation	1.43	1.33	1.45
Median	22.0	20.0	21.0
Minimum	19	18	18
Maximum	23	23	23
Highest grade of school completed			
Grade 10	1 (3.1)	1 (7.7)	2 (4.4)
Grade 11	9 (28.1)	3 (23.1)	12 (26.7)
Grade 12	22 (68.8)	9 (69.2)	31 (68.9)
Completed high school (matric)			
	22 (68.8)	9 (69.2)	31 (68.9)
Tertiary education			
	6 (18.8)	1 (7.7)	7 (15.6)
Relationship status			
Married	0	0	0
Divorced, separated	0	0	0
Single	3 (9.4)	0	3 (6.7)
Widowed	0	0	0
Steady partner, cohabitating	4 (12.5)	1 (7.7)	5 (11.1)
Steady partner, not cohabitating	22 (68.8)	11 (84.6)	33 (73.3)
Casual partner	3 (9.4)	1 (7.7)	4 (8.9)

Forty-two participants (93.3%) reported having active sexual partners, all but one being male. Thirty-three participants (73.3%) had steady non-cohabitating partners, 5 (11.1%) lived with their partners and 4 (8.9%) had casual partners. Three women (6.7%) reported no sexual partner. No one was married, divorced or widowed. During the study, one participant (2.2%) reported a new sexual partner.

Sexual debut in this cohort occurred at a median age of 18 years (range 15–22), and the median lifetime number of sexual partners was 3 (range 1–8). Five women (11.1%) reported experiences with anal sex. During the 6 months prior to the study, participants reported a median of one sexual partner (range 1–3). In the past 30 days before the study, 38 (84.4%) reported having had vaginal sex at a median of two occurrences (range 1–30).

Thirty participants (66.7%) reported at least one previous pregnancy, one experienced a stillbirth (2.2%), six had a spontaneous abortion (13.3%) and no one had a history of elective abortions.

No participant reported use of vaginally inserted agents including vaginal preparations, sexual stimulants or drying agents prior or during the study, and only two (4.4%) used tampons for menstrual hygiene. Per eligibility criteria, all participants used a reliable method of contraception (injectable contraception) for at least one month prior to enrollment. Adverse events (AEs), mostly mild, occurred in 77·8% of all participants with no significant differences between arms. All solicited genitourinary (local) AEs were mild. In both groups, the most common solicited local AEs were abnormal vaginal discharge and vaginal odor, also commonly seen as a symptom of BV. The most common solicited systemic adverse events were abdominal pain or cramps and headaches (20).

Adherence to study product was high. Thirty-six (80%) participants completed all 11 doses of study product over 4 weeks, and 40 (88.9%) completed at least 9 doses. Failure to take study product was mainly due to clinic closure during political unrest and flooding.

Forty-three of 45 participants completed the acceptability questionnaire at the 4 week follow up visit.

Two participants in the LACTIN-V group were lost to follow up when social unrest damaged the clinic and many people in the area were temporarily displaced. Thirty-eight (88.4%) participants agreed or strongly agreed to having been satisfied using the vaginal applicator, 37 (86.0%) to insertion being comfortable, and 41 (95.3%) to the applicator being easy to insert and use. Overall, 74.5% of participants agreed or strongly agreed that they would use the product again, 73.4% in the LACTIN-V group and 76.9% in the placebo group (Table 2).

**Table 2:** Satisfaction with the study product.

*n* (%)	Strongly agree	Agree	Neutral	Disagree	Strongly disagree	Missing data
I was satisfied with the applicator						
LACTIN-V (*N* = 32)	7 (*23.3)*	19 (63.3)	4 (13.3)	0	0	2
Placebo (*N* = 13)	4 (*30.8)*	8 (61.5)	1 (7.7)	0	0	0
All (*N* = 45)	11 (25.6)	27 (62.8)	5 (11.6)	0	0	2
The applicator was comfortable when inserted						
LACTIN-V (*N* = 32)	10 (33.33)	16 (53.3)	3 (10.0)	1 (3.3)	0	2
Placebo (*N* = 13)	2 (15.4)	9 (69.2)	2 (15.4)	0	0	0
All (*N* = 45)	12 (27.9)	25 (58.1)	5 (11.6)	1 (2.3)	0	2
The applicator was easy to insert and use						
LACTIN-V (*N* = 32)	18 (60.0)	11 (36.7)	1 (3.3)	0	0	2
Placebo (*N* = 13)	6 (46.2)	6 (46.2)	1 (7.7)	0	0	0
All (*N* = 45)	24 (55.8)	17 (39.5)	2 (4.7)	0	0	2
I would use the product again						
LACTIN-V (*N* = 32)	11 (36.7)	11 (36.7)	5 (16.7)	2 (6.7)	1 (3.3)	2
Placebo (*N* = 13)	3 (23.1)	7 (53.8)	3 (23.1)	0	0	0
All (*N* = 45)	14 (32.6)	18 (41.9)	8 (18.6)	2 (4.7)	1 (2.3)	2

On a Likert scale of 0–10, participants felt that the study product was effective for their vaginal health (mean 6.7, SD 1.94), comfortable (mean 7.9, SD 1.82) and easy to use (mean 8.7, SD1.50), with no significant difference between groups. The agreement with positive product attributes scored means of ≥6.7, while agreement with negative product attributes scored ≤3.2 (Table 3). Asked whether attributes would make product use harder for them, participants assigned low mean values to these potential impediments: frequency or timing of use (mean 3.2, SD 2.48), vaginal dryness (mean 2.1, SD 2.46), messiness or leakage (mean 2.4, SD 2.74) or partner's disapproval (mean 1.7, SD 2.7).

**Table 3:** Product attributes, Likert scale 0–10.

**POSITIVE PRODUCT ATTRIBUTES: mean** (SD) [range] On a scale of 0 to 10, with 0 being 'not at all' to 10 being 'extremely', I found the product to be:			
	LACTIN-V (N=32)	Placebo (N=13)	All Participants (N=45)
Effective	**6.9** (1.72) [3–10]*	**6.5** (2.44) [0–10]	**6.7** (1.94) [0–10]
Comfortable	**7.8** (2.01) [2–10]	**8.1** (1.32) [6–10]	**7.9** (1.82) [2–10]
Easy to use	**8.8** (1.55) [5–10]	**8.5** (1.45) [6–10]	**8.7** (1.50) [5–10]
**NEGATIVE PRODUCT ATTRIBUTES: mean** (SD) [range] On a scale of 0 to 10, with 0 being 'not at all' to 10 being 'extremely', the following things made the product hard for you to use:			
	LACTIN-V (N=32)	Placebo (N=13)	All Participants (N=45)
Frequency or timing of use	**3.4** (2.53) [0–10]	**2.5** (2.33) [0–6]	**3.2** (2.48) [0–10]
Vaginal dryness	**2.4** (2.69) [0–10]	**1.3** (1.65) [0–4]	**2.1** (2.46) [0–10]
'Messiness' or leakage	**3.0** (2.99) [0–10]	**1.2** (1.54) [0–4]	**2.4** (2.74) [0–10]
Partner's disapproval	**2.1** (3.09) [0–10]	**0.5** (0.66) [0–2]	**1.7** (2.70) [0–10]

* **mean** (SD) [range]

Boldface denotes mean values.

Fourteen (32.5%) participants agreed or strongly agreed that using the product covertly without the partner knowing was an important product feature for them, while 25 (58.2%) disagreed or strongly disagreed, and 4 (9.3%) stayed neutral. When asked whether it was important that the partner approved of the product, 7 (16.3%) agreed or strongly agreed, while 31 (72.1%) disagreed or strongly disagreed, and 5 (11.6%) stayed neutral. Sexual partners were not questioned directly but participants reported on their awareness and reaction of product use. Fifteen of 43 (34.9%) participants stated that their partner was unaware of product use, 2 (4.7%) didn't know the partner's reaction, and 2 (4.7%) didn't have a partner. Among the 24 participants whose partners were aware of product use, 12 (27.9%) characterized their partners’ reaction to the product as positive, 3 (7.0%) as negative and 9 (20.9%) as neutral. No social harms were reported to clinic staff by those participants whose partners had a negative reaction.

## 4 Discussion

In this cohort of young Black South African women, LACTIN-V was found to be highly acceptable, as underscored by at least 74% of participants being satisfied using the vaginal applicator, finding it comfortable and easy to insert and use, and agreeing that they would use the product again, even though very few participants had prior experience with vaginally inserted products including tampons. While other studies using interviews and focus groups report a strong preference for oral administration of LBPs and antibiotics for vaginal health (15, 21), we found that use of the vaginal applicator and use of LACTIN-V were very well accepted. Reported comfort and ease of use of the study product was high, and three quarters of women would use the product again—this finding was not different by study group. These results are comparable with those of participants in other studies of LACTIN-V in U.S. and British cohorts (11, 13, 14), as well as studies of other LBPs promoting vaginal health (15, 16, 22), and similar to the acceptability of vaginal rings and other vaginal products designed for female HIV prevention (23–26). While other studies mentioned leakage or messiness as an impediment for the use of vaginal products (21), our participants did not have this experience.

Almost all participants were sexually active before and during the study. A third of participants stated that their partners were unaware of them using study product; among partners who were aware their reaction was reported as mostly positive or neutral. Only three participants characterized their partners’ reaction as negative. Interestingly, participants overwhelmingly stated that their partner's approval was not important for them (over 70%), and only a third found discreet use of the product without their partner knowing to be an important product feature. These findings are encouraging, especially since the impact of partners’ disapproval on use of vaginal products to promote reproductive health has been associated with low adherence (16, 25).

To our knowledge, this is the first study in Africa assessing a vaginally administered LBP containing a human vaginal *Lactobacillus* strain and classified as an investigational drug. Our recent results on high colonization (up to 69% at the conclusion of the dosing phase) and suppression of CD4+ HIV target cells in the endocervix in this cohort (20) along with high product acceptability support further product development in populations at high risk of HIV acquisition. Limitations of this study include a relatively small sample size, and not including open-ended questions to ascertain broader perceptions about the study product. Other studies of product acceptability have included focus groups and interviews (15), which are better suited to elucidate more in-depth answers to questions about partner support and preferences for product attributes. While these findings on high acceptability of vaginally administered LBPs among young South African women are encouraging, potential social or cultural influences on product acceptability need to be further explored in future studies. These findings among a small cohort of young South African women cannot be generalized to other high-risk populations, such as older women or women in different regions of Africa. Furthermore, future studies need to explore acceptability of longer-term administration of LBPs to assess their suitability as an HIV prevention method.

Planned next steps for the clinical development of LACTIN-V are to conduct a phase 3 study aiming to confirm the effectiveness of LACTIN-V to prevent recurrent BV. Additionally, LACTIN-V is currently in clinical trials in the UK for effectiveness of preventing preterm labor, and a US-based phase 2 study to assess its impact on HPV clearance is starting enrollment in late 2025.

If subsequent clinical trials confirm efficacy, safety, acceptability and colonization of the *Lactobacillus crispatus* CTV-05 strain in different populations around the world, LACTIN-V could become the first LBP of its class with regulatory approval as a drug to prevent vaginal dysbiosis, and potentially contribute to decreasing genital tract inflammation and female HIV acquisition.

## Data Availability

The raw data supporting the conclusions of this article will be made available by the authors, without undue reservation.
